# Cost-effectiveness of seasonal quadrivalent versus trivalent influenza vaccination in the United States: A dynamic transmission modeling approach

**DOI:** 10.1080/21645515.2016.1242541

**Published:** 2016-10-26

**Authors:** Anita J. Brogan, Sandra E. Talbird, Ashley E. Davis, Edward W. Thommes, Genevieve Meier

**Affiliations:** a RTI Health Solutions, Research Triangle Park, NC, USA; b Medical Division, GSK Inc, Mississauga, ON, Canada; c University of Guelph, Guelph, ON, Canada; d GSK Vaccines, Wavre, Belgium

**Keywords:** cost-effectiveness analysis, dynamic transmission model, influenza, quadrivalent influenza vaccine, trivalent influenza vaccine, United States

## Abstract

Trivalent inactivated influenza vaccines (IIV3s) protect against 2 A strains and one B lineage; quadrivalent versions (IIV4s) protect against an additional B lineage. The objective was to assess projected health and economic outcomes associated with IIV4 versus IIV3 for preventing seasonal influenza in the US. A cost-effectiveness model was developed to interact with a dynamic transmission model. The transmission model tracked vaccination, influenza cases, infection-spreading interactions, and recovery over 10 y (2012–2022). The cost-effectiveness model estimated influenza-related complications, direct and indirect costs (2013–2014 US$), health outcomes, and cost-effectiveness. Inputs were taken from published/public sources or estimated using regression or calibration. Outcomes were discounted at 3% per year. Scenario analyses tested the reliability of the results. Seasonal vaccination with IIV4 versus IIV3 is predicted to reduce annual influenza cases by 1,973,849 (discounted; 2,325,644 undiscounted), resulting in 12–13% fewer cases and influenza-related complications and deaths. These reductions are predicted to translate into 18,485 more quality-adjusted life years (QALYs) accrued annually for IIV4 versus IIV3. Increased vaccine-related costs ($599 million; 5.7%) are predicted to be more than offset by reduced influenza treatment costs ($699 million; 12.2%), resulting in direct medical cost saving annually ($100 million; 0.6%). Including indirect costs, savings with IIV4 are predicted to be $7.1 billion (5.6%). Scenario analyses predict IIV4 to be cost-saving in all scenarios tested apart from low infectivity, where IIV4 is predicted to be cost-effective. In summary, seasonal influenza vaccination in the US with IIV4 versus IIV3 is predicted to improve health outcomes and reduce costs.

## Introduction

Seasonal influenza has historically affected an estimated 5–20% of the United States (US) population annually, resulting in approximately 95,000 influenza-related hospitalizations and approximately 8,000 influenza-related deaths on average each year^1-3^ prior to the introduction of quadrivalent influenza vaccines in 2012. Influenza has also had a high annual cost burden, estimated at $10 billion in direct medical costs and $16 billion in lost earnings due to illness and loss of life in the US (2003 US dollars).^4^

Seasonal influenza is best prevented by influenza vaccination,^1^ which is recommended annually for all people aged ≥6 months without contraindications in the US.^5^ A pivotal study showed that vaccination is effective for influenza prevention in children,^6^ and systematic reviews have examined the effectiveness of vaccination across age groups.^7-9^ Despite an upward trend in vaccination rates,^10-14^ overall coverage remains below 50%.^14^

Two main types of influenza vaccines are currently recommended.^5^ Trivalent vaccines (trivalent inactivated influenza vaccines [IIV3s] and a trivalent recombinant influenza vaccine) protect against 2 influenza A strains (H1N1 and H3N2) and one influenza B lineage (Yamagata or Victoria); quadrivalent vaccines (quadrivalent inactivated influenza vaccines [IIV4s] and a quadrivalent live attenuated influenza vaccine [LAIV4]) protect against 2 influenza A strains and both influenza B lineages. The B lineage included in the trivalent vaccines is chosen prior to the start of each influenza season before the actual predominant lineage is known. The decision is based primarily on the predominant lineage from the previous season and in recent years has only been correct about half of the time.^15^ The use of quadrivalent vaccines may therefore reduce seasonal influenza cases, as vaccinated individuals will be protected against both B lineages.

Although a prior cost-effectiveness analysis predicted that IIV4 would be cost-effective at conventional willingness-to-pay thresholds in the US,^16^ the study did not account for the impact of herd protection. The objective of this study was therefore to estimate projected health and economic outcomes associated with IIV4 versus IIV3 for the prevention of seasonal influenza in the US using a dynamic transmission model, in order to better assess the societal value and cost-effectiveness of IIV4.

## Results

### Base case

On average over a 10-year time horizon, influenza vaccination with IIV4 versus IIV3 (vaccinating equal numbers of individuals) is predicted to reduce the discounted annual number of type B cases by 1,973,849 (undiscounted 2,325,644), resulting in 12–13% fewer cases of seasonal influenza and influenza-related complications and deaths (Table 1). Vaccination with IIV4 versus IIV3 is projected to yield 6,005 more life years and 18,485 more quality-adjusted life years (QALYs) annually.

**Table 1.** Ten-year average annual results for the base-case analysis (2012–2022): IIV4 versus IIV3.

Outcomes^a^	IIV3	IIV4	Difference: IIV4–IIV3 (%)
Health outcomes			
Number of people vaccinated	125,479,086	125,479,086	0
Cases of influenza	16,066,932	14,093,083	−1,973,849 (−12.3)
Type A	13,419,031	13,419,031	0
Type B	2,647,901	674,052	−1,973,849 (−74.5)
Influenza-related complications	2,040,773	1,792,822	−247,951 (−12.1)
Influenza-related deaths	10,577	9,181	−1,396 (−13.2)
Life years accrued	251,124,881	251,130,886	6,005 (+0.002)
QALYs accrued	232,293,904	232,312,389	18,485 (+0.008)
Cost outcomes (2013/2014 $)			
Total direct medical costs	16,277,026,239	16,176,911,683	−100,114,557 (−0.6)
Vaccine-related costs	10,565,287,914	11,163,818,974	598,531,060 (+5.7)
Acquisition	1,430,420,828	2,028,951,888	598,531,060 (+41.8)
Administration	9,125,758,120	9,125,758,120	0
Vaccine-related AE management	9,108,966	9,108,966	0
Influenza treatment costs	5,711,738,326	5,013,092,709	−698,645,617 (−12.2)
Inpatient	3,563,010,255	3,129,491,214	−433,519,041 (–12.2)
Outpatient	2,107,708,964	1,847,588,081	−260,120,883 (−12.3)
Non-medically attended	41,019,107	36,013,414	−5,005,693 (−12.2)
Total indirect costs	109,568,786,671	102,615,203,315	−6,953,583,357 (−6.3)
Time lost for vaccination	49,420,957,469	49,420,957,469	0
Caregiver time lost for cases	7,737,050,813	6,682,377,821	−1,054,672,992 (−13.6)
Patient time lost for cases	21,046,749,319	18,491,117,241	−2,555,632,078 (−12.1)
Time lost for influenza-related death	31,364,029,070	28,020,750,783	−3,343,278,287 (−10.7)
Total costs	125,845,812,911	118,792,114,997	−7,053,697,913 (−5.6)
ICERs (2013/2014 $)			
Incremental direct medical costs per QALY gained	−	−	−5,416^b^
Incremental direct medical costs and indirect costs (for time lost for vaccination and for caregiver time lost for cases of influenza) per QALY gained^c^	−	−	−62,472^b^

AE, adverse event; ICER, incremental cost-effectiveness ratio; IIV3, trivalent inactivated influenza vaccine; IIV4, quadrivalent inactivated influenza vaccine; QALY, quality-adjusted life year.

a All health and cost outcomes were discounted to 2012 using an annual discount rate of 3%.^54^

b Negative ICERs shown here indicate that vaccination with IIV4 yielded lower total costs and more QALYs than vaccination with IIV3.

c Costs for patient time lost for cases of influenza and for influenza-related death were not included in the numerator as these time losses are assumed to be captured in the QALY loss estimates; it would therefore be considered double counting to also include the costs of patient time lost in the numerator.^17^

Annual vaccine-related costs are predicted to increase by $599 million (5.7%) with IIV4 versus IIV3 (Table 1). However, this increase is more than offset by an estimated $699 million (12.2%) reduction in influenza treatment costs. Use of IIV4 is therefore projected to result in annual direct medical cost savings of $100 million (0.6%). Overall, IIV4 is predicted to provide more QALYs and lower direct medical costs than IIV3 (incremental cost-effectiveness ratio [ICER]: −$5,416 per QALY gained).

Total indirect costs related to influenza are 6–7-fold higher than direct medical costs (Table 1). When indirect costs are included for time lost for vaccination and caregiver time lost for cases of influenza,^17^ annual total cost savings with IIV4 are predicted to be $1.2 billion (1.6%), with a resulting ICER of –$62,472 per QALY gained. If all indirect costs are included, the total cost savings with IIV4 are estimated to be even greater ($7.1 billion [5.6%]).

### Scenario analyses

Results were generally robust in a variety of clinically relevant scenario analyses. IIV4 was cost-saving versus IIV3 in all scenarios tested apart from low infectivity (i.e., low probability of transmission given an infectious contact), where IIV4 was predicted to be cost-effective (ICER: $19,678 per QALY gained) (Table 2).

**Table 2.** Ten-year average annual scenario analyses results (2012–2022)^a^.

Scenario settings	Cases avoided (IIV4 versus IIV3)	ICER (2013/2014 $ per QALY gained)^b^
Base case	1,973,849	−62,472
Higher infectivity^c^ (β = 0.000529, R_0_ = 3.5)	3,593,862	−50,598
Lower infectivity^c^ (β = 0.000256, R_0_ = 1.69)	563,795	+19,678
Increased duration of natural immunity to type A influenza (Upper 95% CL [2.52 years])	1,973,849	−62,469
Decreased duration of natural immunity to type A influenza (Lower 95% CL [2.38 years])	1,973,849	−62,484
Increased duration of natural immunity to type B influenza (Upper 95% CL [15.44 years])	1,834,046	−60,710
Decreased duration of natural immunity to type B influenza (Lower 95% CL [13.94 years])	2,130,249	−64,058
Increased natural cross-protection (Upper 95% CL [Type A = 52.2%, Type B = 52.0%])	1,938,301	−61,964
Decreased natural cross-protection (Lower 95% CL [Type A = 44.3%, Type B = 48.3%])	2,012,049	−62,993
Increased amplitude of season variation factor (Upper 95% CL [0.472])	2,103,013	−64,232
Decreased amplitude of season variation factor (Lower 95% CL [0.443])	1,857,467	−60,605
Increased probability of selecting the correct B lineage (Upper 95% CL [71.2%])	1,925,708	−62,269
Decreased probability of selecting the correct B lineage (Lower 95% CL [65.3%])	1,973,849	−62,472
Fixed vaccine coverage projections^d^ (at 2012 levels)	2,489,427	−70,708
Increased percentage of children receiving 2 doses of IIV3 or IIV4^e^	1,973,849	−62,368
Increased inpatient costs per case (base case +50%)	1,973,849	−74,198
Decreased inpatient costs per case (base case −50%)	1,973,849	−50,745
Lower vaccine administration cost (nurse setting; $20.06 visit + $25.08 administration)	1,973,849	−62,472

CL, confidence limit; ICER, incremental cost-effectiveness ratio; IIV3, trivalent inactivated influenza vaccine; IIV4, quadrivalent inactivated influenza vaccine; QALY, quality-adjusted life year.

a All health and cost outcomes were discounted to 2012 using an annual discount rate of 3%.^54^

b ICER includes direct medical costs, the cost for time lost for vaccination, and the cost for caregiver time lost for cases of influenza. Negative ICERs shown here indicate that vaccination with IIV4 yielded lower total costs and more QALYs than vaccination with IIV3.

c Infectivity was varied from the base-case value of β = 0.000287 (R_0_ = 1.9).^22^

d Vaccination coverage values for 2012–2022 were assumed to remain fixed at 2012–2013 values^14^ rather than following a projected increase over time.

e Using age-specific estimates of percentages of children <9 years old who received 2 doses of vaccine.^11^

### Model validation

Model validation tested a variety of extreme scenarios and yielded differences in model results between IIV3 and IIV4 that followed expected patterns. For example, health outcomes were equivalent for IIV3 and IIV4 when vaccine efficacy against the B lineage not included in IIV3 was increased to equal efficacy against the B lineage included in IIV3.

## Discussion

In this dynamic transmission modeling study that assessed the cost-effectiveness of IIV4 versus IIV3 in the US, our base-case analysis predicted that vaccination with IIV4 would result in 12–13% fewer cases of seasonal influenza and influenza-related complications and deaths. Although IIV4 is more expensive than IIV3, higher vaccination costs are more than offset by reductions in influenza treatment costs, resulting in lower total direct medical costs and improved health outcomes. When indirect costs are included, the economic savings with IIV4 are predicted to be even greater.

The model results were most sensitive to changes in infectivity. Any vaccination program is likely to be more cost-effective if the virus is more readily transmitted. However, even when low infectivity was examined, IIV4 exhibited an ICER less than commonly accepted US willingness-to-pay thresholds of $50,000 per QALY gained^18^ or 1–3 times the US gross domestic product per capita ($46,405–$139,216 [2014] per QALY gained).^19,20^ All other scenario analyses predicted that IIV4 would be cost-saving versus IIV3.

A probabilistic sensitivity analysis (PSA) was not conducted – in line with recommendations from the International Society for Pharmacoeconomics and Outcomes Research-Society for Medical Decision Making (ISPOR-SMDM) Task Force^21^ – as many of the parameters in a transmission model are highly correlated. This correlation is critical to ensure reasonable results, but is difficult to preserve in the context of a PSA.^21^

The current transmission model was adapted from one by Thommes et al.^22^ that was used to predict the effect of switching from a targeted to a universal influenza immunization program in Canada. Only one other study has used a transmission model to predict the effects of using IIV4 versus IIV3.^23^ This German individual-based simulation model predicted that IIV4 use would prevent 4.3% of influenza cases versus IIV3 but did not examine cost-effectiveness. This smaller clinical benefit compared with our study is likely explained by differences in model design and input parameters (e.g., lower vaccine coverage in Germany).

Several static economic models have compared vaccination with IIV4 versus IIV3 (or IIV3/trivalent live attenuated influenza vaccine [LAIV3]) in various countries (US,^16^ United Kingdom,^24,25^ Canada,^26^ Hong Kong^27,28^), but none accounted for herd protection. The models all predicted that IIV4 versus IIV3 would result in reductions in influenza cases, hospitalizations, and deaths and predicted positive ICERs (more QALYs, higher costs), although whether IIV4 was cost-effective varied across countries and age groups considered. The US study^16^ reported an ICER of $90,301 per QALY gained for IIV4 versus IIV3, whereas the current study predicted IIV4 to be cost saving (more QALYs, lower costs). This difference is principally explained by our use of a transmission model that inherently accounts for herd protection.

### Strengths and limitations

One of the key benefits of dynamic transmission models versus static models is that transmission models account for benefits to the entire population resulting from vaccination of a portion of the population (i.e., herd protection). This analysis used a transmission model with accompanying cost-effectiveness calculations to account for the full health-economic impact of seasonal influenza vaccination and therefore provides reliable cost and health outcome estimates. The transmission model included the evolution of the 2 dominant type A strains and the 2 B lineages each year and modeled long-term time horizons using appropriate input parameter projections. The 10-year time horizon captured the inter-seasonal variability of influenza and thus provided more robust model results than could be achieved by considering any given single influenza season. Life years, QALYs, and earnings lost due to influenza-related death were estimated only within the 10-year model time horizon and were attributed in the year they would have occurred. The model conservatively did not account for losses due to influenza-related death beyond the 10-year model time horizon.

This analysis has the typical limitations of pharmacoeconomic analyses. The model predicted the dynamics of seasonal influenza and the resulting health and economic outcomes based on the best available input data. Published estimates were not available for all parameters; therefore, calibration and linear interpolation were utilized to estimate some of the parameters. Because IIV4 was introduced relatively recently (2012/2013 season), model parameters were based on published literature and observed influenza patterns before its introduction. The model is intended to help predict outcomes associated with the use of IIV4, but information on the real-world impact of IIV4 is limited. As there are no head-to-head studies of overall IIV4 versus IIV3 efficacy, this study combined information on circulating strains, vaccine composition, and strain-specific efficacies based on studies that found that IIV4s have non-inferior immunogenicity to IIV3s for the shared strains/lineages and superior immunogenicity for the additional B lineage.^29,30^ Based on evidence of clinical similarity between influenza types^31,32^ and a lack of robust contrary type-specific data, all influenza-related parameters in the economic analysis (e.g., complication rates, costs) were assumed equivalent for type A and B influenza. Also due to a lack of robust data, the percentage of influenza cases managed in the emergency department (ED) was set to zero. Finally, this study was not intended to assess the use of LAIVs or to predict or model pandemic influenza.

### Generalizability

The model developed for this study was populated with US-specific data and calibrated to observed influenza patterns in the US; therefore, the results are applicable to the overall US population only. Cost and health outcome results are presented as annual averages over the 10-year model time horizon (2012/2013 to 2021/2022 influenza seasons) and do not represent projections for any specific influenza season or for influenza seasons beyond 2022. The model structure is generalizable, and adaptation of country-specific inputs and recalibration would yield results applicable to other countries.

### Conclusions

Model findings predict that IIV4 would result in substantially fewer cases of seasonal influenza, fewer influenza-related complications and deaths, and more QALYs accrued versus IIV3. Use of IIV4 is predicted to result in higher vaccination costs than IIV3, but this increase is predicted to be more than offset by reductions in direct medical costs, with further cost savings predicted when indirect costs are included. IIV4 is therefore predicted to be cost-saving compared with IIV3 (more QALYs gained at a lower cost). These results were consistent in all scenarios tested except low infectivity, where IIV4 is predicted to be cost-effective compared with IIV3. Therefore, a shift from IIV3 to IIV4 for seasonal influenza vaccination in the US is predicted to result in better health outcomes and lower total costs.

## Methods and materials

### Model structure

**Figure 1.**
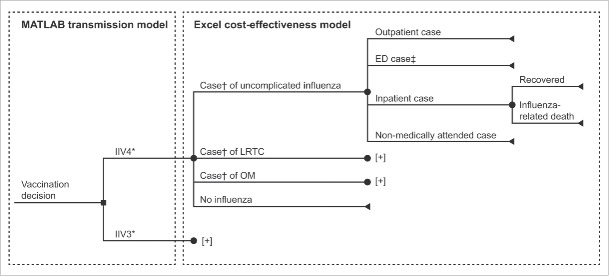
Simplified model structure for cost-effectiveness of IIV4 versus IIV3 (the full structure can be found in Thommes et al.^22^). *A proportion of individuals were vaccinated with either IIV3 or IIV4 according to the vaccine coverage parameters shown in Figure. 2. †Case of influenza type A or B. ‡ED cases were set to zero due to a lack of robust data. [+] indicates clinical pathway is the same as above. ED, emergency department; IIV3, trivalent inactivated influenza vaccine; IIV4, quadrivalent inactivated influenza vaccine; LRTC, lower respiratory tract complication; OM, otitis media.

A cost-effectiveness model (Microsoft Excel 2010) was developed to interact with an adapted dynamic transmission model (MATLAB R2012a). The transmission model tracked vaccination, influenza infection and recovery, and infection-spreading interactions. The cost-effectiveness model utilized these results to estimate influenza-related complications and all associated costs and health outcomes. Fig. 1 shows all possible influenza vaccination, infection, complication, and treatment pathways for the modeled population. The time horizon for the analysis was 10 y (2012–2022), which allowed annual averages that account for seasonal variation to be calculated.

**Figure 2.**
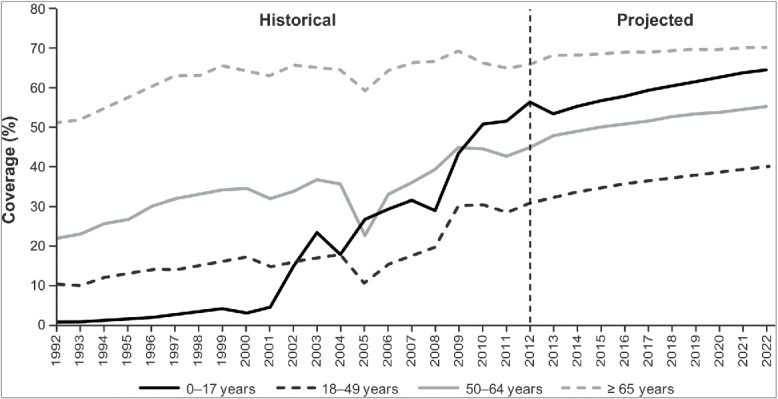
Historical^10-14^ and projected influenza vaccine coverage by age group. Historical vaccine coverage data were available through 2012, and data were not available for every year for children aged <18 years; therefore, vaccination coverage estimates for future years and for years without historical estimates were projected using regression techniques and available historical data. Exponential and logarithmic functions were tested, and best-fit functions were selected for each age group.

### Transmission model

The transmission model was adapted from a susceptible–infected–recovered–vaccinated (SIRV) compartmental model developed by Thommes et al.^22^ The modeled population included all US residents, stratified into 100 yearly age groups, and was tracked over time with annual birth and death rates applied. A proportion of the population received vaccination with either IIV3 or IIV4 during the 3-month vaccination program beginning October 1 of each year. Infectivity and recovery were modeled for 2 influenza A strains (the 2 dominant strains each season rather than specific strains) and 2 B lineages each season. A contact matrix specified the mean duration of daily contact between a member of any given age group and members of all age groups.^33^ The model inherently accounted for herd protection as vaccinated individuals were less likely to become infected and were therefore less likely to transmit the virus to unvaccinated individuals via contact. Natural cross-protection was modeled between A strains by assuming a percentage of individuals who recovered from one A strain infection developed immunity to the other A strain (similarly for B lineages). A 20-year warm-up period (1992–2012) initialized the model to the starting conditions of the first year of analysis.

### Cost-effectiveness model

The cost-effectiveness model assessed the health economic impact of vaccinating the US population with IIV3 or IIV4 from a US societal perspective. The analysis included vaccine-related costs (acquisition, administration, and adverse event [AE] management); influenza-related medical costs; and indirect costs (wages lost due to vaccination and influenza). Utility values (ranging from 0 [dead] to 1 [perfect health]) were used to estimate QALY losses due to influenza. Health and cost outcomes for IIV3 and IIV4 were used to calculate incremental outcomes including ICERs.

### Input parameters

#### Population inputs

Age and gender distribution data for the modeled US population in 1992 (i.e., the start of the warm-up period) were taken from the 1990 US census.^34^ Birth rates by the age of the mother^35^ and death rates^36^ were based on historical estimates for 1992–2012 and were projected to 2013–2022 using regression techniques. Best-fit functions were selected among the exponential and logarithmic functions tested. Other characteristics of the modeled population, such as health status and access to care, were assumed to be reflective of the overall US population.

#### Vaccination inputs

In the model, people of all ages were eligible for vaccination annually. (Scenarios with varying eligibility by age are examined in the Supplementary Methods and Results.) Historical vaccination coverage – with IIV3 – increased during the 20-year warm-up period (1992–2012) (Fig. 2).^10-14^ Vaccination coverage during the 2012–2022 period – with IIV3 or IIV4 (same coverage in both arms) – was also projected to increase (Fig. 2).

Vaccine acquisition costs for children were based on a weighted average of public and private costs (2014 US dollars)^37^ (based on a personal communication [unpublished data] on the percentage of children eligible for the publicly funded Vaccines for Children program [2010 VFC Eligible Children. Centers for Medicare & Medicaid Services. VFC Comparison of Regional Maximum Rate for Vaccine Administration to Current State Rate. “2010PES_final_8/18/09”]); vaccine acquisition costs for adults were based on private wholesale acquisition costs^37^ (Table 3). The cost of vaccine administration included a clinic visit plus administration by either a physician or a nurse,^38^ in equal proportions. Individuals generally received one dose of influenza vaccine; however, the Advisory Committee on Immunization Practices recommends that children aged 6 months through 8 years receive 2 doses (administered ≥4 weeks apart) if they have not previously received an influenza vaccine.^5^ The model assumed that each year, all vaccinated children aged <1 year received 2 doses and therefore incurred doubled costs for vaccine acquisition, administration, and vaccine-related caregiver productivity losses. This assumption was tested in a scenario analysis. Indirect costs related to time for vaccination were included in the model, assuming patients or caregivers lost 2 hours from work or usual activities for vaccination,^39^ resulting in lost wages of $38.80 (in 2013 US dollars^40^).

**Table 3.** Vaccine-related inputs.

	Age range (years)							
	0–2	3–4	5–17	18–49	50–64	65–74	≥75	Sources
Vaccine acquisition cost per dose ($)^a^								^37^
IIV3, public cost	7.75	7.75	7.75	7.52	7.52	7.52	7.52	
IIV3, private sector cost	10.69	11.30	11.30	11.90	11.90	11.90	11.90	
IIV4, public cost	15.29	13.65	13.65	12.22	12.22	12.22	12.22	
IIV4, private sector cost	18.62	16.15	16.15	16.15	16.15	16.15	16.15	
Vaccine administration cost ($)								^38^
Visit cost^b^	46.57	46.57	46.57	46.57	46.57	46.57	46.57	
Administration cost^c^	25.08	25.08	25.08	25.08	25.08	25.08	25.08	
Cost per vaccine-related AE ($)^d^								^39,41,43^
Anaphylaxis	3,864	3,864	3,864	592	636	629	629	
Guillain-Barre syndrome	33,427	33,427	33,427	86,237	86,237	86,237	86,237	
Vaccine efficacies (IIV3 and IIV4) (%)								^8,9,16,44-46^
Type A	59.0	59.0	59.0	61.0	61.0	58.0	58.0	
Type B included in vaccine	66.0	66.0	77.0	77.0	73.0	69.0	66.0	
Type B not included in vaccine	44.0	44.0	52.0	52.0	49.0	47.0	44.0	

AE, adverse event; IIV3, trivalent inactivated influenza vaccine; IIV4, quadrivalent inactivated influenza vaccine.

a The median public and median private cost for IIV3 and IIV4 indicated for each age group are shown. The model used the weighted average of public and private costs (2014 US dollars), assuming public costs were incurred for 63.2% of those aged <1 year, 55.8% for 1–2 years, 50.9% for 3–6 years, 41.7% for 7–17 years, and 0% for adults (personal communication [unpublished data]: 2010 VFC Eligible Children. Centers for Medicare & Medicaid Services. VFC Comparison of Regional Maximum Rate for Vaccine Administration to Current State Rate. “2010PES_final_8/18/09”).

b Visit cost based on the average of CPT codes 99213 (physician, $73.08) and 99211 (nurse, $20.06).^38^

c Vaccine administration cost based on CPT codes 90460 (children) and 90471 (adults).^38^

d Inflated to 2013 US dollars.^43^

The model included costs and QALY losses for vaccine-related AEs. Incidences of anaphylaxis and Guillain-Barre syndrome were 1 per 4 million and 1 per million, respectively.^39,41^ QALYs lost per AE were 0.020 and 0.141, respectively.^39,42^ Costs per AE varied by age (Table 3).^39,41,43^

Vaccine efficacies against A strains (58–61%) and the B lineage(s) included in the vaccine (66–77%) varied by age and were estimated as previously described^16^ based on various reviews and meta-analyses^8,9,44-46^ (Table 3). To account for cross-protection from vaccination, vaccine efficacies against the B lineage not included in IIV3 were 44–52% (Table 3). Vaccine effectiveness for IIV3 and IIV4 varied each season based on age-specific vaccine efficacies, the age distribution of the population, the B lineage selected for IIV3, and the proportion of circulating influenza caused by type B overall and by each type B lineage. The mean duration of vaccine-induced immunity was 1 y.^22^

#### Influenza-related inputs

Evidence suggests that type A and B influenza are clinically similar,^31,32^ and robust contrary type-specific data are limited. Therefore, all influenza-related inputs, except natural cross-protection and duration of natural immunity, were assumed to be the same for type A or B influenza (Table 4). All people with influenza were assumed to be equivalently infectious, even if they were asympomatic (33.1% of cases).^22,47^ Given an infectious contact, the probability of transmission (β) was 0.000287 per minute of contact (seasonally adjusted R_0_ = 1.9).^22^ The rate of recovery (i.e., loss of infectiousness rather than of symptoms) was 0.25 per day, calculated based on a 4-day average duration of infectiousness.^48^

**Table 4.** Influenza-related inputs (for type A and type B influenza).

	Age range (years)					
	0–4	5–17	18–49	50–64	≥65	Sources
Complicated/uncomplicated influenza distribution (%)						
Influenza with LRTC^a^	2.12	1.02	13.19	17.30	20.94	^49,50^
Influenza with OM	11.88	3.13	0.79	0.35	0.21	^49,50^
Uncomplicated influenza	86.01	95.85	86.02	82.35	78.85	Complement of above
Influenza with LRTC: treatment setting distribution (%)						
Outpatient^b^	47.87	35.16	35.95	41.60	72.24	Calculated from Molinari et al.^4^
ED	0	0	0	0	0	Assumption (no data)
Inpatient^c^^,^^d^	0.82	0.34	1.24	2.84	6.18	^49,50^
Non-medically attended	51.31	64.50	62.82	55.56	21.58	Complement of above^4^
Influenza with OM or uncomplicated influenza: treatment setting distribution (%)						
Outpatient^b^	47.87	35.16	35.95	41.60	72.24	Calculated from Molinari et al.^4^
ED	0	0	0	0	0	Assumption (no data)
Inpatient^c^^,^^e^	0.82	0.34	0.46	0.82	2.21	^49,50^
Non-medically attended	51.31	64.50	63.59	57.58	25.55	Complement of above^4^
Inpatient cases resulting in death (%)^f^	0.28	1.67	2.14	6.94	27.79	Calculated from Molinari et al.^4^
QALYs lost per case						<18 years;^39^ ≥18 years calculated
Influenza with LRTC, inpatient	0.076	0.076	0.0115	0.0115	0.0093	from Gold et al.^51^ and Lee et al.^52^
Influenza with LRTC, other	0.046	0.046	0.0115	0.0115	0.0093	
Influenza with OM, all	0.042	0.042	0.0052	0.0052	0.0036	
Uncomplicated influenza, inpatient	0.076	0.076	0.0115	0.0115	0.0093	
Uncomplicated influenza, other	0.005	0.005	0.0052	0.0052	0.0036	
Medical care costs per case ($)^g^						^4,43^
Outpatient^b^	269	220	307	490	518	
Inpatient^b^	20,831	25,565	33,326	40,890	20,268	
Non-medically attended (over-the-counter medication)	4.29	4.29	4.29	4.29	4.29	
Patient/caregiver time lost per case (days)						^4,53^
Outpatient	1.38	1.38	1.15	2.66	5.05	
Inpatient	9.13	9.13	13.34	16.63	15.56	
Non-medically attended	1.0	0.5	0.5	0.5	1.0	

ED, emergency department; LRTC, lower respiratory tract complication; OM, otitis media; QALY, quality-adjusted life year.

a For ages 0–17 years, complications rates were based on pneumonia diagnoses only.^49^ For ages ≥18 years, complication rates were based on pneumonia and respiratory diagnoses.^50^

b Weighted average of high-risk and non-high risk individuals.^4^

c For ages 0–17 years, hospitalization rates were based on “any hospitalization” (regardless of complications).^49^

d For ages ≥18 years, hospitalization rates were based on pneumonia and respiratory hospitalizations.^50^

e For ages ≥18 years, hospitalization rates were based on all-cause hospitalizations minus pneumonia and respiratory hospitalizations.^50^

f Values were converted from mortality rates among all cases of influenza^4^ to conditional probabilities among inpatient cases of influenza. Deaths among cases that are treated outside the hospital (or not medically attended) were assumed to be negligible.

g Inflated to 2013 US dollars.^43^

An individual with influenza could have uncomplicated influenza or influenza with a lower respiratory tract complication (LRTC) or otitis media (OM).^49,50^ Patients with symptomatic influenza (whether complicated or not) could be non-medically attended or treated as outpatients or inpatients; inpatients were at risk for influenza-related death.^4,49,50^ Patients with asymptomatic influenza did not have complications and were non-medically attended. All parameters varied by age (Table 4).

QALYs lost per case of influenza or influenza-related complication for children were taken from Prosser et al.^39^ (Table 4). For adults, QALYs lost were calculated by applying reduced utility values for the assumed duration of illness. Utilities for healthy adults were 0.92 (age 18–64 years) or 0.84 (≥65 years),^51^ and utilities for influenza cases were 0.65 (uncomplicated) or 0.50 (LRTC or hospitalized).^52^ These reduced utilities were applied for 7 d (uncomplicated) or 10 d (LRTC or hospitalized).^52^ A utility value of zero was used for cases of influenza-related death.

Costs for all outpatient and inpatient cases were taken from a previously published economic model and US claim analysis by Molinari et al. (2007).^4^ The mean cost per outpatient case included influenza-related physician visits and prescription medications obtained within 3 d of the visit; the mean cost per inpatient case included influenza-related hospitalizations, physician visits, and prescription medications obtained 2 weeks prior to hospitalization through 30 d post-discharge.^4^ Costs were inflated to 2013 US dollars.^43^

Costs for productivity losses due to illness among patients or caregivers of patients <16 years of age were calculated by multiplying the median US daily wage ($155.20 in 2013^40^) by the number of days lost from work or usual activities (Table 4). For all adults and non-medically-attended children, time loss estimates were taken from the study by Molinari et al.^4^ For medically-attended children, time loss estimates for caregivers were taken from a US caregiver interview study.^53^ Costs for productivity losses due to influenza-related mortality were calculated by multiplying years of life lost (within the remaining model time horizon of 10 years) by the median US annual wage,^40^discounted to 2012 at 3% per year.^54^ These costs were included for people of all ages, assuming initiation of productivity at age 16 years.

#### Calibrated inputs

Observed characteristics from recent influenza seasons were used to calibrate parameter values for which literature/public sources were not available. The calibration process is summarized in the Supplementary Methods and Results. Mean durations of natural immunity to influenza were estimated to be 2.5 y (type A) and 14.7 y (type B), similar to values from Thommes et al.^22^ Calibration found natural cross-protection to be 48.3% between A strains and, coincidentally, 48.3% between B lineages. The amplitude of season variation factor, multiplying the force of infection, was 0.457. The probability that the B lineage selected for IIV3 became the predominant B lineage that season was estimated at 68.2%.

### Model outcomes

Health outcomes included the number of people vaccinated, cases of influenza and influenza-related complications (LRTC and OM) and deaths, and life years and QALYs accrued. Cost outcomes included vaccine costs (acquisition, administration, management of vaccine-related AEs), influenza-related medical costs, indirect costs (time lost for vaccination, caregiver/patient time lost for influenza, premature mortality), total costs, and ICERs. All outcomes were discounted to 2012 at an annual rate of 3%, per US guidelines;^54^ undiscounted influenza cases are also reported to quantify the public health impact of vaccination.

### Analyses

#### Base case

After a 20-year warm-up, the 10-year base-case analysis compared vaccination with IIV4 or IIV3 starting with the 2012–2013 influenza season and ending in 2021–2022. Annual averages were calculated over the 10-year time horizon for each model outcome.

#### Scenario analyses

A number of clinically relevant scenario analyses were undertaken to assess the robustness of the results. These scenarios included increased and decreased infectivity (β),^55-57^ duration of natural immunity to type A and B influenza, natural cross-protection, amplitude of seasonal variation factor, probability of selecting the correct B lineage, and inpatient costs. Other scenarios tested were lower vaccine administration costs (all doses administered by a nurse); more children receiving 2 vaccine doses, as reported by parents/guardians in the 2010–2011 National Flu Survey;^11^ and lower vaccine coverage during 2012–2022 (fixed at 2012–2013 levels^14^).

In line with recommendations from the ISPOR-SMDM Task Force,^21^ a PSA was not conducted.

#### Model validation

Model validation was undertaken to confirm the model's response to various extreme scenarios. For example, vaccine efficacy against the B lineage not included in IIV3 was set to zero or set equal to the efficacy of the B lineage included in IIV3; the probability of selecting the correct B lineage to include in IIV3 was set to 1.0; and vaccine coverage was set to 0% and 100%.

## Supplementary Material

KHVI_A_1242541_Supplementary_material.zip

## Abbreviations

AE: adverse event
ED: emergency department
ICER: incremental cost-effectiveness ratio
IIV3: trivalent inactivated influenza vaccine
IIV4: quadrivalent inactivated influenza vaccine
ISPOR-SMDM: International Society for Pharmacoeconomics and Outcomes Research – Society for Medical Decision Making
LAIV3: trivalent live attenuated influenza vaccine
LAIV4: quadrivalent live attenuated influenza vaccine
LRTC: lower respiratory tract complication
OM: otitis media
PSA: probabilistic sensitivity analysis
QALY: quality-adjusted life year
SIRV: susceptible–infected–recovered–vaccinated
US: United States
