# Supplementary material for: Cost-effectiveness of seasonal quadrivalent versus trivalent influenza vaccination in the United States: A dynamic transmission modeling approach
Source: Hum Vaccin Immunother. 2016 Oct 26;13(3):533–42. doi: 10.1080/21645515.2016.1242541 (PMC5360116; doi:10.1080/21645515.2016.1242541)
Supplement: KHVI_A_1242541_Supplementary_material.zip [file khvi-13-03-1242541-s001.zip › KHVI_A_1242541 Supplementary material.pdf]

Supplementary Methods and Results

Article title: Cost-effectiveness of seasonal quadrivalent versus trivalent influenza vaccination in the United States: A dynamic transmission modeling approach

Authors: Anita J. Brogan, Sandra E. Talbird, Ashley E. Davis, Edward W. Thommes, Genevieve Meier

Calibration methods

Calibration was used to estimate values for parameters for which literature or public sources were not available. Calibration was conducted using methodology similar to that described by Thommes et al.[1]

First, specific influenza season target outcomes were identified, and acceptable target ranges representing feasible values for each outcome during the calibration period (2000–2009) were specified using published literature. Target outcomes were: (a) annual attack rates;[2] (b) annual percentage of cases that are type A;[3] and (c) annual percentage of type B cases that match the lineage included in IIV3[4] (Table S1). Bootstrapping[5] was used to define acceptable target ranges for (b) and (c) because confidence intervals (CIs) were not available in the source documents.

Second, specific model parameters were identified for calibration. The parameters were: mean durations of natural immunity to type A and B influenza; natural cross-protection between A strains and between B lineages; amplitude of season variation factor, multiplying the force of infection; and the probability that the B lineage selected for IIV3 at the beginning of the vaccination period (based on the most prevalent lineage at the time) becomes the most prevalent B lineage during the influenza season (Table S2). For each calibration parameter, an initial range of values, referred to as the “prior range” (Table S2) was defined, which included all remotely feasible values for each calibration parameter. The prior range for each parameter was tightened during calibration to only those values that resulted in realistic values for the target outcomes.

Once the target outcomes and calibration parameters had been defined, the calibration was conducted to find appropriate values for the calibration parameters. The following steps were used to complete the calibration procedure:

- 10,000 calibration sets were formed using Latin Hypercube sampling.[6] Each set included a sampled value of each calibration parameter from the prior ranges.

- A model run was completed using each calibration set.
- After each run, outputs were averaged across seasons and analyzed to confirm whether the average model outputs were all within the acceptable target ranges (shown in Table S1). Parameter sets were accepted if all outputs were within the allowable target ranges, but rejected if not.
- After all of the runs had been completed, a posterior distribution was formed for each calibration parameter using the accepted parameter sets. The final value of each calibrated parameter was defined as the mean of the corresponding posterior distribution.
- One final calibration run was conducted using the final calibrated parameter set to confirm that it resulted in an acceptable model run.

Calibration results

From the 10,000 calibration runs, 209 were found to be acceptable. The mean accepted value for each target outcome and the corresponding 95% CI among all accepted calibration runs are summarized in Table S1.

Table S1. Acceptable target ranges and mean accepted values for the three seasonal influenza target outcomes.

Target outcome	Acceptable target range	Source	Mean accepted value (95% CI)[a]
Annual influenza attack rates (%)			
Children (0–17 years)	14.9–24.5	Turner et al., 2003[2]	16.5 (16.3–16.7)
Non-elderly adults (18–64 years)	2.9–12.6	Turner et al., 2003[2]	11.2 (11.1–11.3)
Elderly adults (≥ 65 years)	2.9–12.6	Turner et al., 2003[2]	5.1 (4.9–5.2)
Annual influenza cases that are type A (%)	70.3–87.7[b]	CDC, 2014[3]	80.0 (79.3–80.7)
Annual type B cases matched by IIV3 vaccine (%)	28.5–76.7[b]	Reed et al., 2012[4]	43.0 (41.8–44.2)

CI, confidence interval.

[a] Standard CIs were calculated assuming a normal sampling distribution and utilized the mean and standard deviation of acceptable calibration runs for each target outcome.

[b] Bootstrapping[5] was used to define these acceptable target ranges because CIs were not available in the source documents.

The mean accepted parameter values shown in Table S2 were utilized for the economic analyses.

Table S2. Prior ranges, sources, and mean accepted values and 95% CIs for each calibration parameter.

Calibration parameter	Prior range	Source of prior range	Mean (95% CI) accepted value[a]
Mean duration of natural immunity to type A influenza (years)	1–6	Personal communication (unpublished data)[b]	2.45 (2.38–2.52)
Mean duration of natural immunity to type B influenza (years)	5–35	Personal communication (unpublished data)[b]	14.69 (13.94–15.44)
Natural cross-protection between A strains (%)	0–100	Maximum allowable range	48.3 (44.3–52.2)
Natural cross-protection between B lineages (%)	0–100	Maximum allowable range	48.3 (44.6–52.0)
Amplitude of season variation factor, multiplying the force of infection	0.2–0.6	Approximately ±50% the value (0.43) from Vynnycky et al.[7]	0.457 (0.443–0.472)
Probability of selecting the correct B lineage[c] (%)	0–100	Maximum allowable range	68.2 (65.3–71.2)

CI, confidence interval; SD, standard deviation; IIV3, trivalent inactivated influenza vaccine.

[a] Standard CIs were calculated assuming a normal sampling distribution and utilized the mean and SD of acceptable calibration runs for each calibration parameter.

[b] 2010 VFC Eligible Children. Centers for Medicare & Medicaid Services. VFC Comparison of Regional Maximum Rate for Vaccine Administration to Current State Rate. "2010PES_final_8/18/09".

[c] Probability that the B lineage selected for IIV3 at the beginning of the vaccination period (based on the most prevalent lineage at the time) becomes the most prevalent B lineage during the season.

Validation of calibration results

Once the calibration process was completed and the values of the calibration parameters were updated in the model, the number of influenza cases and the timing of the modeled seasonal influenza peaks were validated. Figure S1 compares the amplitude and timing of seasonal influenza peaks during the calibration period with Centers for Disease Control and Prevention (CDC) FluView data.[8] CDC FluView data[8] provide reported influenza cases whereas

the model includes all cases. In the graphs, modeled cases are therefore scaled using a reporting fraction of 66.9%.[9] On average, the amplitude and timing of seasonal influenza peaks was similar. The timing does not match perfectly because the model does not account for anomalous influenza seasons.

Figure S1. Comparison of weekly type A and type B influenza cases. Modeled cases were scaled using a reporting fraction of 66.9%[9] for comparison with CDC reported cases.[8]

Type A

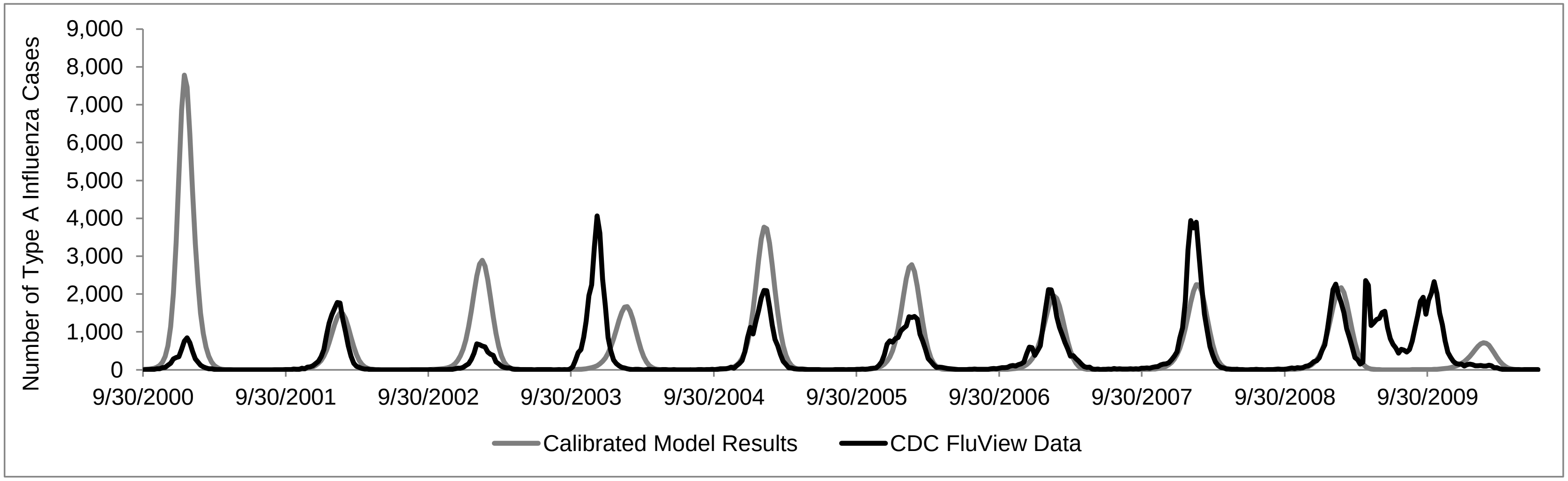

Type B

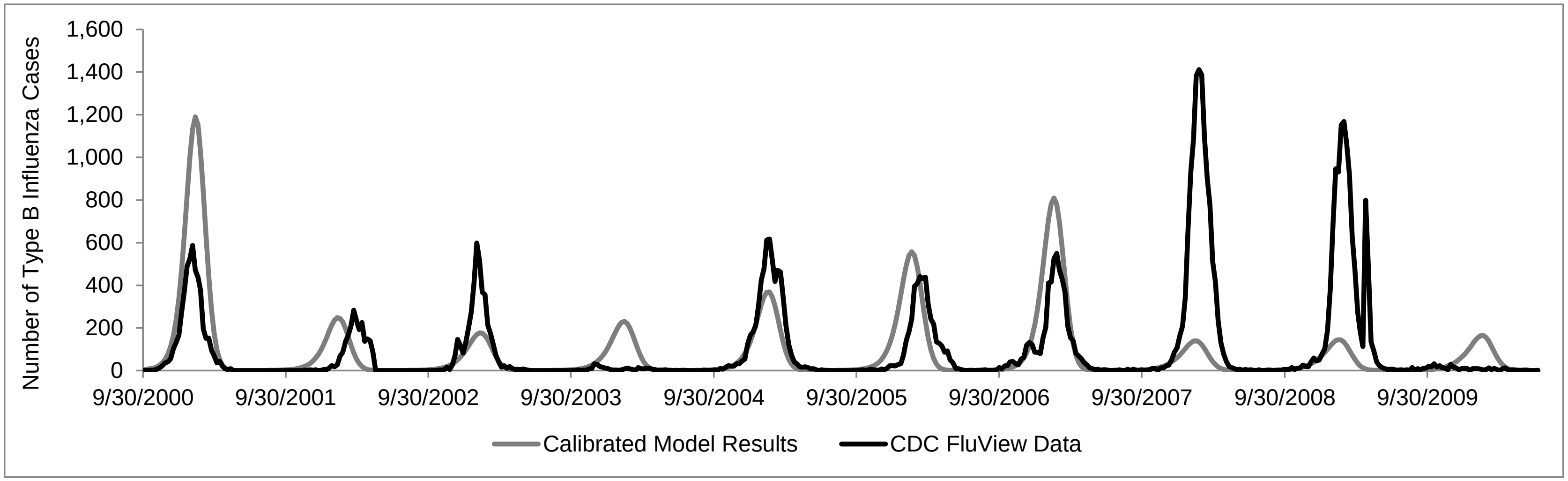

Additional Age-specific Vaccination Scenario Analyses

Additional hypothetical vaccination scenario analyses were conducted to study the impact of vaccinating only specific subpopulations: 0-4 years, 5-17 years, 18-49 years, 50-64 years, and 65+ years. In each scenario, individuals eligible for vaccination were vaccinated annually at the same coverage levels used in the base-case analysis. Vaccinating specific age groups in isolation is not a likely real-world strategy; however, these analyses provide further insight into the costs and benefits of vaccinating specific age groups.

IIV4 was found to be cost-saving versus IIV3 in all scenarios analyzed (Table S3). The scenario analysis results differed in QALYs gained and cost savings for a variety of reasons, most notably because the total number of individuals vaccinated varied by age group and because each age group interacted differently within its own age group and across the other age groups.

Table S3. Ten-year average annual results for age-specific vaccination scenario analyses (2012-2022): IIV4 versus IIV3

	**Eligible for vaccination**				
**Incremental (IIV4 vs. IIV3) Outcomes[a]**	**0–4 years**	**5–17 years**	**18–49 years**	**50–64 years**	**65+ years**
Health outcomes					
Number of people vaccinated[b]	9,481,043	25,332,716	36,720,755	25,992,715	28,762,613
Cases avoided	443,074	745,701	971,695	238,099	391,755
QALYs gained	4,802	6,839	8,937	3,008	9,786
Cost outcomes (2013/2014 $)					
Total direct medical costs	−71,371,086	−90,082,966	−168,388,033	−15,336,167	−116,970,824
Total costs including select indirect costs[c]	−425,393,445	−619,292,237	−634,593,856	−68,784,255	−217,270,355
ICER (2013/2014 $[d] per QALY gained)	−88,587	−90,553	−71,007	−22,867	−22,202

ICER, incremental cost-effectiveness ratio; IIV3, trivalent inactivated influenza vaccine; IIV4, quadrivalent inactivated influenza vaccine; QALY, quality-adjusted life year.

[a] All health and cost outcomes were discounted to 2012 using an annual discount rate of 3%.[10]

[b] The same number of people were vaccinated in both model arms with either IIV3 or IIV4.

[c] Select indirect costs included the cost for time lost for vaccination and the cost for caregiver time lost for cases of influenza.

[d] The numerator of the ICER included direct medical costs and select indirect costs.

References

1. Thommes EW, Chit A, Meier GC, Bauch CT. Examining Ontario's universal influenza immunization program with a multi-strain dynamic model. Vaccine 2014; 32:5098-117.
2. Turner D, Wailoo A, Nicholson K, Cooper N, Sutton A, Abrams K. Systematic review and economic decision modelling for the prevention and treatment of influenza A and B. Health Technol Assess 2003; 7:iii-iv, xi-xiii, 1-170.
3. Centers for Disease Control and Prevention (CDC). Influenza vaccination coverage estimates by State, HHS Region, and the United States, National Immunization Survey (NIS) and Behavioral Risk Factor Surveillance System (BRFSS), 2010-11 through 2012–13 seasons. Available at http://www.cdc.gov/flu/fluvaxview/reports/reporti1213/trends/index.htm, accessed April 7, 2014.
4. Reed C, Meltzer MI, Finelli L, Fiore A. Public health impact of including two lineages of influenza B in a quadrivalent seasonal influenza vaccine. Vaccine 2012; 30:1993-8.
5. Singh K, Xie M. Bootstrap: A Statistical Method. Available at http://www.stat.rutgers.edu/home/mxie/rcpapers/bootstrap.pdf, accessed October 22, 2013.
6. Iman RL. Latin Hypercube Sampling. Encyclopedia of Quantitative Risk Analysis and Assessment. 2008.
7. Vynnycky E, Pitman R, Siddiqui R, Gay N, Edmunds WJ. Estimating the impact of childhood influenza vaccination programmes in England and Wales. Vaccine 2008; 26:5321-30.
8. Centers for Disease Control and Prevention (CDC). WHO/NREVSS National Influenza Positive Tests reported to CDC during 2000-2010 influenza seasons. Available at http://gis.cdc.gov/grasp/fluview/fluportaldashboard.html, accessed February 18, 2014.
9. Carrat F, Vergu E, Ferguson NM, Lemaitre M, Cauchemez S, Leach S, Valleron AJ. Time lines of infection and disease in human influenza: a review of volunteer challenge studies. Am J Epidemiol 2008; 167:775-85.
10. Gold MR, Siegel JE, Russel LB, Weinstein MC. Cost-Effectiveness in Health and Medicine. Oxford University Press, 1996.
